# Supplementary material for: Predictive Value of Circulating miRNAs in Lymph Node Metastasis for Colon Cancer
Source: Genes (Basel). 2021 Jan 27;12(2):176. doi: 10.3390/genes12020176 (PMC7912296; doi:10.3390/genes12020176)
Supplement: Supplementary file 1 [file genes-12-00176-s001.pdf]

Supplementary Materials

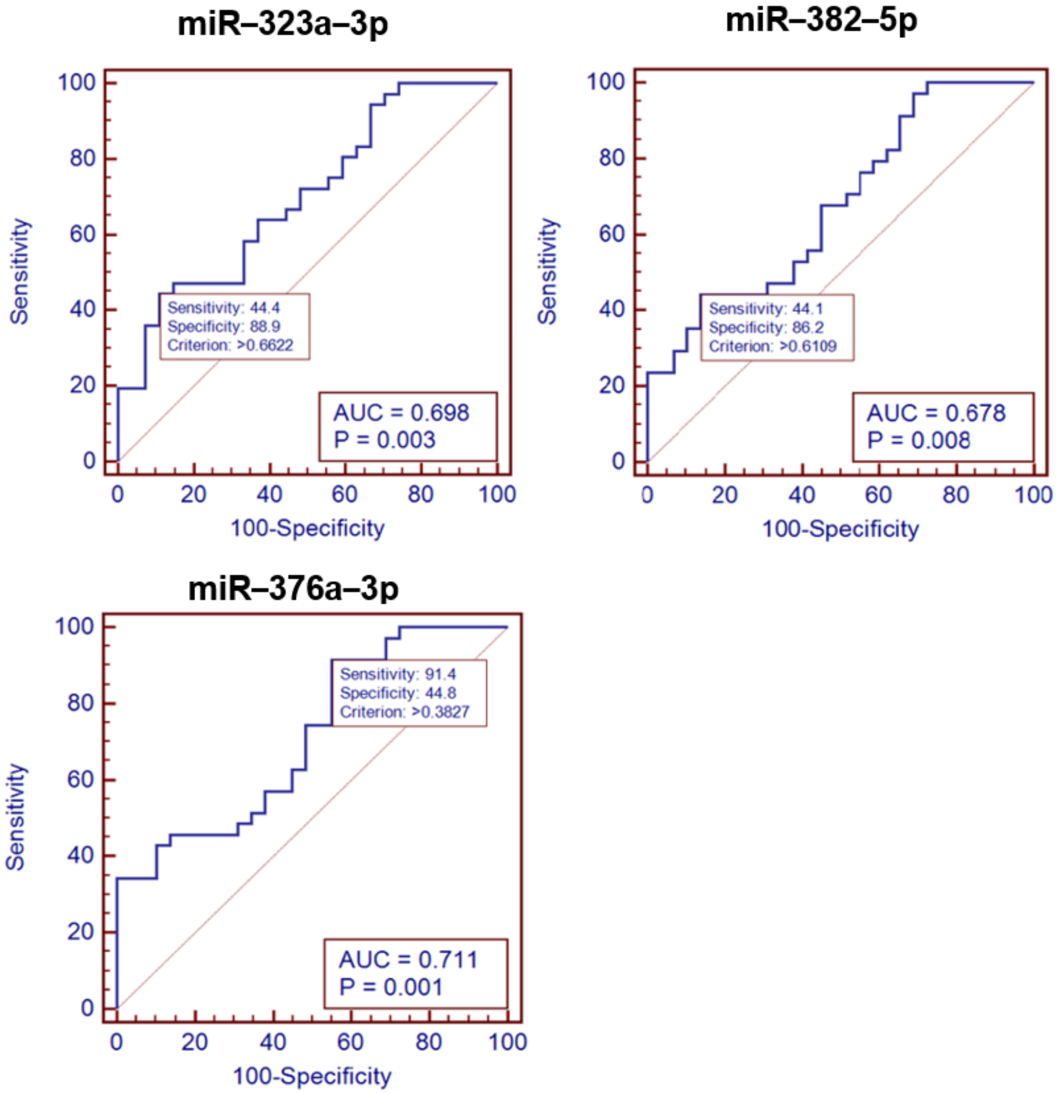

Figure S1. ROC curves of the three miR levles for determining the presence of LN metastasis. The area under the ROC curve (AUC) for LN metastasis was highest for miR-376a-3p (0.711), followed by miR-323a-3p (0.698) and miR-382-5p(0.678).
